# Resident Medical Officers and Matrons

**Published:** 1913-11-29

**Authors:** 


### The Hospital

**The Workers' Newspaper of Administrative Medicine and Institutional Life, Administration, National Insurance and Health.**

No. 1431, Vol. LV. SATURDAY, NOVEMBER 29, 1913. PRICE ONE PENNY.

### RESIDENT MEDICAL OFFICERS AND MATRONS.

The appointment of a matron always entails a very great responsibility on hospital committees, and this responsibility is increased rather than decreased in the cases of small hospitals. The choice of the right candidate presupposes a knowledge of womankind in excess of that possessed by the average hospital committee. Often some comparatively trivial point decides the election committee in favour of one particular candidate, and lucky indeed is the institution which can claim the services of a well-balanced, efficient matron. The consequences which sooner or later will follow the installation of an unsuitable woman in the post of matron are felt even more severely in the smaller institutions than in the larger hospitals. In those institutions which have only one, or at the most two, resident medical officers, it is easy to see that the whole harmony of the place depends on the cordial co-operation between the matron and the resident medical officer. In these cases the matron's position necessarily looms larger in a more limited sphere, while she is often brought more closely into touch with many of the ultimate details of the institution's daily work.

Notwithstanding all that may be said as to the last word resting with the resident medical officer, who is responsible for the welfare of the patients, for whom in point of fact the institution was built, it is necessary to appreciate that in actual practice the matron *qua* administration is usually the superior officer. The resident medical officer is often but the deputy of the honorary visiting staff, and moreover usually only a bird of passage into the bargain; the matron, on the other hand, has almost unlimited power over her subordinates, and these include not only the sisters and nurses, but the domestic staff as well—that is, practically everybody in the place except the resident medical officer. That the successful control of such a staff is a task demanding special qualities is a self-evident proposition, but it is not, perhaps, sufficiently recognised how rare it is to find a matron possessing more than half the desirable qualities. Only those persons—and they are not numerous—who have had the opportunity of serving in several hospitals are in a position to realise the truth of this statement, and also to appreciate how often it happens that otherwise really excellent women are found to fail when they are placed in positions of considerable authority. This observation is certainly true as far as the nursing profession is concerned, whatever may be the experience in other walks of life.

The office of matron has been gradually evolved, and its status has improved with that of the whole nursing profession during the last fifty years, but nevertheless the fact remains that a matron is still neither more nor less than a head nurse. In the smaller hospitals, with which we are particularly concerned, the matron not rarely performs certain nursing duties either as a rule or as a matter of emergency. It is only in the largest institutions that the title of lady superintendent has any justification, and yet it is not uncommon to find this in use in comparatively small hospitals. Why is it that many matrons fail to differentiate clearly between the inevitable hardships of a nurse's life and those that are distinctly avoidable? Many of the discomforts from which nurses suffer in certain institutions are perpetuated simply because the matron in her early days had to put up with the results of indifference and bad management, and she thinks that the succeeding generations of nurses should also be subjected to these hardships. We are not referring to questions of discipline or training; in fact, as far as discipline is concerned this type of matron is often lenient in that respect, a policy which accentuates other discomforts. It is curious to find that if there is one avoidable grievance which is more often met with than another it is the question of the meals supplied to the nurses. This is a matter which is rarely organised on a satisfactory basis, even at the present day, though it must be admitted that, generally speaking, nurses' meals are now less unsatisfactory than they were ten years ago. The lack of care bestowed on such an important feature of hospital life was truly astonishing, and when it is borne in mind how well care is repaid in this matter by the increased efficiency of the work done, and by the improved general tone of the workers, the neglect of this fundamental principle in hospital management becomes doubly difficult to understand. See our recent article on "Economy," November 15 (page 177).

In many ways a matron may become most autocratic and yet receive no check. The fault here lies in the general failure of committees to come into actual live contact with the working of the hospital. Neither the house surgeon nor the matron attends their meetings, except on specific occasions, and as for the nurses they have no means of approaching the committee except through the matron herself. Very often the matron dismisses nurses without so much as bare intimation to the committee. Except on the rarest occasions the outside world hears nothing of the internal troubles or dissensions of the hospital world. The resident medical officer can soon get another appointment; the sisters put up with things till they have served a decent time; and nurses, who are bound for so many years, must just stick to it. Very often nothing may be noticed except an unusual frequency of new faces among the wardmaids and servants—for they are in the easier position of being able to leave *ad libitum*. This permanence, contrasted with the brief tenure of medical appointments, is the explanation of the lasting defects that sometimes persist unchecked in hospital administration.

